# Development and Validation of a Risk Model for Breast Cancer–Related Lymphedema

**DOI:** 10.1001/jamanetworkopen.2020.24373

**Published:** 2020-11-11

**Authors:** Jennifer Yin Yee Kwan, Petra Famiyeh, Jie Su, Wei Xu, Benjamin Yin Ming Kwan, Jennifer M. Jones, Eugene Chang, Kenneth W. Yip, Fei-Fei Liu

**Affiliations:** 1 Department of Radiation Oncology, University of Toronto, Toronto, Ontario, Canada; 2 Institute of Medical Science, University of Toronto, Toronto, Ontario, Canada; 3 Research Institute, Princess Margaret Cancer Centre, Toronto, Ontario, Canada; 4 Biostatistics Division, Princess Margaret Cancer Centre, Toronto, Ontario, Canada; 5 Department of Diagnostic Radiology, School of Medicine, Queen’s University, Kingston, Ontario, Canada; 6 Cancer Rehabilitation and Survivorship Program, Princess Margaret Cancer Centre, Toronto, Ontario, Canada; 7 Department of Medical Biophysics, University of Toronto, Toronto, Ontario, Canada; 8 Radiation Medicine Program, Princess Margaret Cancer Centre, Toronto, Ontario, Canada

## Abstract

**Importance:**

Approximately 1 in 5 patients with breast cancer who undergo axillary lymph node dissection will develop lymphedema. To appropriately triage and monitor these patients for timely diagnosis and treatment, robust risk models are required.

**Objective:**

To evaluate the prognostic value of mammographic breast density in estimating lymphedema severity.

**Design, Setting, and Participants:**

This prognostic study collected data from July 16, 2018, to March 3, 2020, from the electronic health records of patients of the Cancer Rehabilitation and Survivorship Program at the Princess Margaret Cancer Centre in Toronto, Ontario, Canada. Participants included women who had completed curative treatment for a first diagnosis of breast cancer and who were referred to the program. Also included were a sample of patients in the general breast oncology population who were receiving follow-up care at the center during the same period but who were not referred to the program. All patients attended follow-up appointments at the Princess Margaret Cancer Centre from January 1, 2016, to May 1, 2018. The cohort was randomly split 2:1 to group patients into a training cohort and a validation cohort.

**Exposures:**

Participant demographic and clinical characteristics included age, sex, body mass index (BMI), medical history, cancer characteristics, and cancer treatment.

**Main Outcomes and Measures:**

Spearman correlation coefficient between measured and predicted volume of lymphedema was calculated. Area under the curve (AUC) values were generated for predicting the occurrence of at least mild lymphedema (volume, >200 mL) and severe lymphedema (volume, >500 mL) at the time of initial lymphedema diagnosis.

**Results:**

A total of 373 female patients (median [interquartile range] age, 52.3 [45.9-60.1] years) were eligible for this analysis. Multivariate linear regression identified 3 patient factors (age, BMI, and mammographic breast density), 1 cancer factor (number of pathological lymph nodes), and 1 treatment factor (axillary lymph node dissection) as independent prognostic variables. In validation testing, Spearman correlation revealed a statistically significant moderate correlation (coefficient, 0.42; 95% CI, 0.26-0.56; *P* < .001) between measured volume and predicted volume of lymphedema. The AUC values were 0.72 (95% CI, 0.60-0.83) for predicting the occurrence of mild lymphedema and 0.83 (95% CI, 0.74-0.93) for severe lymphedema.

**Conclusions and Relevance:**

This prognostic study found that patients with low breast density appeared to be at a higher risk of developing severe lymphedema. The finding suggests that by combining breast density with established risk factors a multivariate linear regression model could be used to predict the development of lymphedema and provide volumetric estimates of lymphedema severity in patients with breast cancer.

## Key Points

**Question:**

Can breast density shown on the diagnostic mammogram accurately estimate the severity of upper extremity lymphedema that develops after treatment in a patient with breast cancer?

**Findings:**

In this prognostic study of 373 women with breast cancer, a multivariate linear regression model that used mammographic breast density, body mass index, age, number of pathological lymph nodes, and axillary lymph node dissection performed well in predicting the development of severe lymphedema (volume of >500 mL).

**Meaning:**

The findings of this study suggest that mammographic breast density can be used as a prognostic factor for lymphedema risk and provide volumetric estimates for lymphedema severity.

## Teaser

This prognostic study describes a multivariate linear regression model that combines mammographic breast density with patient, cancer, and treatment factors to identify the possible posttreatment volume of lymphedema in patients with breast cancer.

## Introduction

Approximately 1 in 5 patients with breast cancer who undergo an axillary lymph node dissection (ALND) experiences secondary lymphedema as a surgical complication.^1^ Risk of lymphedema nearly doubles when surgical treatment is combined with radiotherapy^2^ or chemotherapy.^3^ In lymphedema, the lymphatic drainage is damaged, resulting in swelling and deformity of skin and adipose tissues. Patients experience psychosocial morbidity, decreased mobility, and medical complications (eg, infection,^4^ additional malignant neoplasm^5^) associated with up to a 7-fold increase in medical costs.^6^ More than 70% of lymphedema cases develop within the first year after surgical treatment.^7^ With 1 in 8 women having breast cancer and 90% of these women surviving for longer than 5 years after treatment,^8^ the number of individuals with lymphedema is substantial.

Lymphedema becomes increasingly challenging to treat over time because of the development of progressive fibrosis during late stages of this condition. Understanding who is at greatest risk of lymphedema will facilitate monitoring, earlier disease diagnosis, and early initiation of therapies to decrease disease morbidity. Currently, most lymphedema risk models are based on cancer and treatment risk factors, yet these features do not fully account for the risk. Improved risk modeling that incorporates the underlying patient-specific biological drivers of this condition is required for a more accurate and personalized risk assessment.

To date, the main biological factor identified is high body mass index (BMI [calculated as weight in kilograms divided by height in meters squared]) (relative risk, 5.5).^1^ Given that as many as 1 in 2 patients with cancer has obesity, this risk factor is of great concern.^9^ Body mass index has been identified to be an independent risk factor for lymphedema development regardless of cancer treatment.^10^ Biologically, BMI is a gross estimate of the body adiposity of an individual based on height and weight. Across different studies, BMI has demonstrated a high variation in the significance and effect size with regard to lymphedema risk.^11^ Overall, BMI is a mediocre surrogate for fat deposition and high lipid levels, which cause lymphatic degeneration, leakiness, and edema.^12,13^

The main study question was, can breast density shown on the diagnostic mammogram be used to accurately estimate the severity of upper extremity lymphedema that develops after treatment in a patient with breast cancer? We hypothesized that a more direct measurement of fat deposition would have a greater prognostic value than BMI. A common diagnostic examination that all patients with breast cancer undergo is the mammogram, a radiographic image of the breasts that reflects tissue composition based on density. The Breast Imaging Reporting and Data System (BI-RADS) scoring system is used by radiologists to classify breast density from lowest density (radiolucent fat) to highest density (radiopaque epithelial and connective tissue). In general, the BI-RADS score assists with mammogram interpretation given that examinations of patients with dense breasts have decreased sensitivity for cancer detection.

The current study repurposed the standard mammography to measure body adiposity^14,15,16^ as reflected in breast density. To our knowledge, this study is the first to evaluate mammographic breast density for its prognostic value in predicting lymphedema occurrence and severity. We combined this breast density variable with other known patient, cancer, and treatment factors to use as a novel model for early risk assessment of lymphedema.

## Methods

### Study Design and Population

This prognostic study received approval from the institutional review board of the University Health Network. Because the study presented minimal risk and used routine clinical data, it received a waiver of informed consent from the institutional review board. We followed the Transparent Reporting of a Multivariable Prediction Model for Individual Prognosis or Diagnosis (TRIPOD) reporting guideline.^17^

The study included women who had completed curative treatment for a first diagnosis of breast cancer and were referred to the Cancer Rehabilitation and Survivorship (CRS) Program at the Princess Margaret Cancer Centre in Toronto, Ontario, Canada, for survivorship issues such as lymphedema, fatigue, function and mobility issues, and neurocognitive changes. Patients were excluded from the study if they had recurrent or metastatic breast cancer diagnoses or were undergoing palliative treatment. All patients attended follow-up appointments at the Princess Margaret Cancer Centre from January 1, 2016, to May 1, 2018.

The study cohort had greater than 90% power to detect a correlation (correlation coefficient, 0.3; α = .05).^18^ Starting January 1, 2016, we consecutively included, as an independent comparative sample, those patients in the general breast oncology population who were attending follow-up appointments during the same period but who were not referred to the CRS Program. This addition to the cohort enabled us to assess the differences in characteristics between patients who were referred and those who were not referred to the CRS Program. Sample size was calculated for greater than 80% power to detect a small difference between groups (effect size, 0.4; α = .05).^18,19^

### Data Collection

Data were collected from July 16, 2018, to March 3, 2020. Patient data from the CRS Program were prospectively entered by the health care team into the electronic health record using a standard dictation template. From the electronic health record, we extracted the following data: demographic characteristics (age and sex), medical history (vascular disease and immunological disorder), physical examination measurements (height and weight), cancer diagnosis (mammogram report and pathology), primary surgical cancer treatment (lumpectomy or mastectomy), lymph node surgical procedure (sentinel lymph node biopsy or ALND and number of nodes removed and tested positive for breast cancer), chemotherapy administration, radiation treatment, and duration of follow-up.

Age was calculated as the age from date of birth through the date of surgical cancer treatment. The sex of all participants in this study was female given that breast cancer–related lymphedema most commonly occurs in women; less than 1% of breast cancers occur in men.^20^ Vascular disorders included hypercholesterolemia, hypertension, and diabetes. Immunological disorders included any inflammatory or immune-related diseases (eg, rheumatoid arthritis and lupus). Mammographic breast density was reported by licensed radiologists and recorded as a numerical value from 1 to 4 based on the BI-RADS scoring system.^21,22^ Data were collected from the most recent date of mammogram to the initial cancer consultation. Time to diagnosis of lymphedema was calculated as the time from surgical cancer treatment through the first lymphedema clinic appointment at the Princess Margaret Cancer Centre. Lymphedema was documented as the increased volume (milliliters) in the lymphedema-affected arm vs the contralateral unaffected arm.

### Measurement of Lymphedema

A standardized protocol was used for limb measurements, which were obtained by occupational, physical, and registered massage therapists. For patients with a lymphedema diagnosis, limb measurements were obtained at 7 standard points, and volume was calculated from the circumferential measurements.^23^ Patients without a diagnosis of lymphedema were assigned a nil volume outcome.

### Statistical Analysis

Random sampling was performed to group patients into a training cohort and a validation cohort using a 2:1 split. Baseline patient, disease, and treatment characteristics were presented using mean (with SD) and median (with minimum-maximum range or interquartile range [IQR]) for continuous variables and frequency (with %) for categorical variables. Group comparisons were performed using a nonparametric Kruskal-Wallis test or an unpaired, 2-tailed *t* test for continuous variables or χ^2^ test for categorical variables.

Univariate and multivariate ordinary least-squares linear regression models were used to evaluate the association of patient, disease, and treatment characteristics with lymphedema volume. Estimates with 95% CIs and *P* values were calculated. For binary variables, lack of a characteristic was assigned to be the reference level (eg, no history of vascular disease was the reference for calculating the estimate for history of vascular disease). A multivariate linear regression model was selected from variables with *P* < .10 on univariate analysis. Model selection was a stepwise regression, with a statistical significance of a 2-sided *P* < .05 to stay or enter the model. Body mass index and mammographic breast density values were imputed by mean numbers. A sensitivity analysis was conducted without imputation; the estimates were similar.

The Kolmogorov-Smirnov test was applied to confirm comparable probability distributions between training cohort residuals and validation cohort residuals (*P* > .05). Correlation testing was applied to the training and validation cohorts between estimated and observed values as well as correlations between different prognostic metrics. Pearson correlation was used for variables of normal distribution, and Spearman correlation was used for all other associations. Receiver operating characteristic curves were generated to predict lymphedema occurrence using a bootstrap technique (n = 1000 replicates). Kaplan-Meier event-free probability graphs were constructed from the initial surgical cancer treatment date to the occurrence of lymphedema or last follow-up appointment. Events were demarcated by occurrence of lymphedema. Two groups were defined from the predicted volume of lymphedema by applying the model equation. Cox proportional hazard ratios (HRs) were calculated with reference to the risk group with a lower predicted volume. Kaplan-Meier event-free probability graphs were also generated for risk groups defined by breast density.

Statistical analyses were performed with R, version 3.1.2 (R Project for Statistical Computing), and SAS, version 9.4 (SAS Institute Inc). Prism, version 8.4.2 (GraphPad) was used to create the correlation graphs and heatmaps. Area under the curve (AUC) graphs were generated using the fbroc package^24^ in R, version 3.5.1 (R Project for Statistical Computing). Three of us (J.Y.Y.K., J.S., and W.X. ) conducted all data analyses from April 7 to August 31, 2020.

## Results

**Figure 1.**
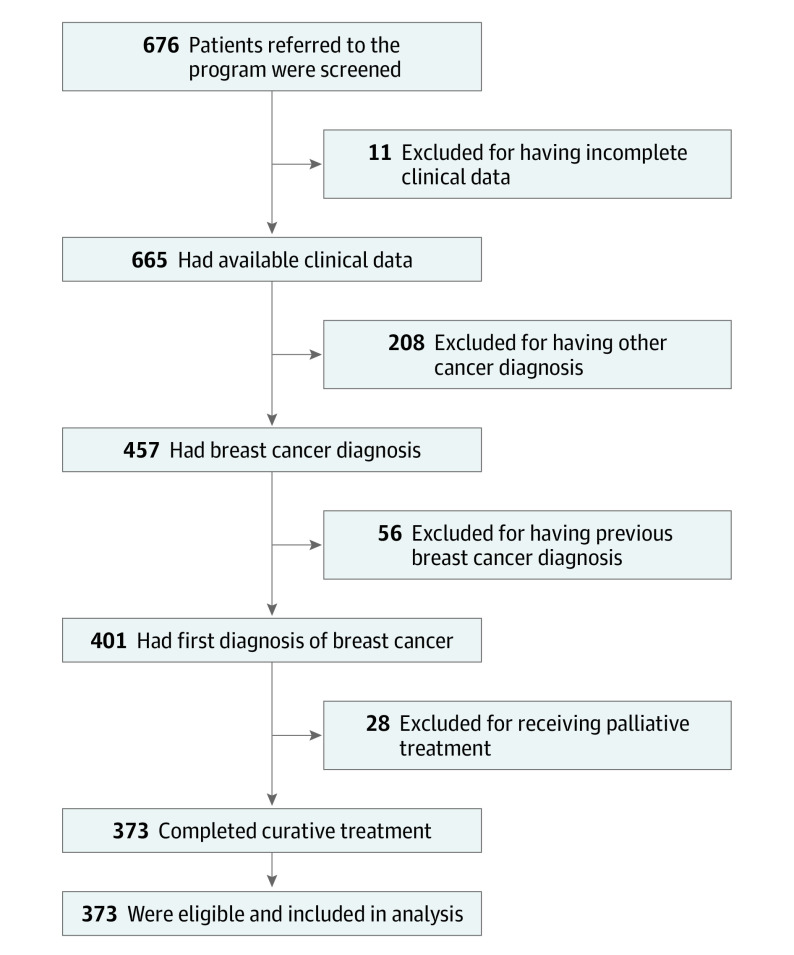
Patient Eligibility

Among the 676 patients referred to the CRS Program in follow-up medical care between January 1, 2016, and May 1, 2018, 373 female patients (55.2%) were eligible for this prognostic study based on a first diagnosis of breast cancer and treatment with curative intent (Figure 1). These women had a median (IQR) age of 52.3 (45.9-60.1) years, and their median (IQR) length of follow-up from date of cancer diagnosis was 1.1 (0.6-2.5) years. Patients without lymphedema had a median (IQR) follow-up of 1.8 (1.0-3.1) years. The training (n = 247) and validation (n = 126) cohorts were comparable in baseline characteristics. For example, the mean (SD) BMI was 26.9 (6.0) in the training cohort and 27.8 (5.9) in the validation cohort, and the mean (SD) mammographic breast density was 2.5 (0.8) in the training cohort and 2.6 (0.7) in the validation cohort (Table 1).

**Table 1.** Baseline Characteristics of Study Population

Characteristic	No. (%)		*P* value^a^
	Training cohort (n = 247)	Validation cohort (n = 126)	
**Patient factors**			
Age, y			
Mean (SD)	53.4 (10.2)	53.2 (11.0)	.57
Median (range)	52.5 (26.4-83.6)	51.5 (31.8-80.9)	
History of vascular disease	71 (28.7)	48 (38.1)	.08
History of immune disorder	42 (17.0)	18 (14.3)	.55
History of surgical seroma	78 (31.6)	38 (30.1)	.81
BMI			
Mean (SD)	26.9 (6.0)	27.8 (5.9)	.17
Median (range)	25.6 (11.0-48.0)	26.3 (16.8-48.5)	
Missing data	32	29	
Pretreatment hemoglobin level			
Mean (SD)	133.1 (13.3)	132.9 (11.8)	.62
Median (range)	135 (24-156)	134 (78-162)	
Missing data	77	40	
Mammographic breast density			
Mean (SD)	2.5 (0.8)	2.6 (0.7)	.63
Median (range)	3 (1-4)	3 (1-4)	
Missing data	43	28	
**Cancer factors**			
Breast cancer diagnosis			
DCIS	7 (2.8)	7 (5.6)	.22
IDC	211 (86.1)	106 (84.1)	
LCIS	1 (0)	0	
ILC	21 (8.6)	13 (10.3)	
Other	6 (2.4)	0	
Missing data	1	0	
ER positive	192 (80.3)	102 (83.6)	.48
Missing data	8	4	
PR positive	170 (71.4)	85 (70.2)	.81
Missing data	9	5	
*ERBB2* positive^b^	39 (16.9)	24 (20.5)	.46
Missing data	16	9	
Cancer stage			
0	5 (2.0)	4 (3.2)	.83
I	56 (22.8)	26 (20.6)	
II	117 (47.6)	63 (50.0)	
III	68 (27.6)	33 (26.2)	
Missing data	1	0	
Tumor size, mm			
Mean (SD)	26.9 (24.8)	28.4 (25.0)	.77
Median (range)	22 (0-150)	23 (0-115)	
Missing data	5	1	
Pathological lymph nodes, No.			
Mean (SD)	2.3 (4.2)	2.8 (6.3)	.95
Median (range)	1 (0-28)	1 (0-57)	
Missing data	2	2	
**Treatment factors**			
Mastectomy	124 (50.2)	57 (45.2)	.38
Axillary lymph node dissection	134 (54.3)	58 (46.0)	.15
Lymph nodes removed, No.			
Mean (SD)	11.8 (9.0)	10.6 (9.7)	.15
Median (range)	11 (0-42)	7 (1-63)	
Missing data	1	3	
Chemotherapy	177 (71.7)	92 (73.0)	.81
Breast/chest irradiation	203 (82.2)	106 (84.1)	.67
Regional nodal irradiation	144 (58.3)	79 (62.7)	.44
Follow-up time			
Mean (SD)	2.1 (2.5)	1.8 (2.4)	.03
Median (range)	1.2 (0.1-16.9)	1 (0.2-14.5)	
Lymphedema arm volume, mL			
Mean (SD)	129.1 (223.8)	106.0 (188.2)	.21
Median (range)	40.6 (0-1634)	21.4 (0-1172)	
Limited, ≤200 mL, No. (%)	196 (79.4)	102 (81.0)	
Mild, range: >200 mL to ≤500 mL, No. (%)	34 (13.8)	19 (15.1)	
Severe, >500 mL, No. (%)	17 (6.9)	5 (4.0)	

Abbreviations: BMI, body mass index (calculated as weight in kilograms divided by height in meters squared); DCIS, ductal carcinoma in situ; ER, estrogen receptor; IDC, invasive ductal carcinoma; ILC, invasive lobular carcinoma; LCIS, lobular carcinoma in situ; PR, progesterone receptor.

^a^ Group comparisons were performed using a nonparametric Kruskal-Wallis test for continuous variables or χ^2^ test for categorical variables.

^b^ *ERBB2* (formerly *HER2*).

Univariate linear regression analysis of the training cohort data demonstrated that several variables were associated with lymphedema volume. These variables were 4 patient factors (age, history of vascular disease, BMI, and mammographic breast density), 3 cancer factors (invasive lobular carcinoma pathology, higher cancer stage, and number of pathological lymph nodes), and 3 treatment factors (ALND, number of lymph nodes removed, and regional nodal irradiation) (Table 2).

**Table 2.** Univariate and Multivariate Linear Regression for Lymphedema Volume

Variable	Estimate (95% CI)	*P* value
**Univariate characteristic**^a^		
Patient factors		
Age	5.99 (3.35 to 8.64)	<.001
History of vascular disease	100.53 (40.04 to 161.02)	.001
History of immune disorder	–66.74 (–140.7 to 7.23)	.08
History of surgical seroma	53.6 (–6.18 to 113.38)	.08
BMI	12.83 (7.94 to 17.71)	<.001
Pretreatment hemoglobin level	–2.38 (–4.82 to 0.06)	.056
Mammographic breast density	–83.05 (–121.96 to – 44.15)	<.001
Cancer factors		
Breast cancer diagnosis		.24
DCIS	0 [Reference]	NA
IDC	103.33 (–64.53 to 271.19)	.23
LCIS	111.56 (–355.54 to 578.65)	.64
ILC	199.90 (9.21 to 390.60)	.04
Other	153.06 (–90.03 to 396.14)	.22
ER positive	21.10 (–51.29 to 93.50)	.57
PR positive	23.29 (–40.61 to 87.20)	.48
*ERBB2* positive	–8.94 (–87.48 to 69.61)	.82
Higher stage (III vs 0-II)	103.66 (42.70 to 164.61)	<.001
Tumor size, mm	0.82 (–0.31 to 1.96)	.15
No. of pathological lymph nodes	16.35 (10.04 to 22.66)	<.001
Treatment factors		
Mastectomy	–5.83 (–61.75 to 50.09)	.84
Axillary lymph node dissection	158.58 (106.08 to 211.08)	<.001
No. of lymph nodes removed	7.85 (4.89 to 10.82)	<.001
Chemotherapy	52.05 (–9.65 to 113.76)	.10
Breast/chest irradiation	36.04 (–36.90 to 108.99)	.33
Regional nodal irradiation	82.49 (26.72 to 138.25)	.004
**Multivariate characteristic**^b^		
Patient factors		
Age	3.73 (1.29 to 6.18)	.003
BMI	10.10 (5.51 to 14.70)	<.001
Mammographic breast density	–37.34 (–73.98 to –0.70)	.046
Cancer factors		
No. of pathological lymph nodes	12.65 (6.59 to 18.71)	<.001
Treatment factor		
Treated with axillary lymph node dissection	99.30 (47.61 to 150.99)	<.001

Abbreviations: BMI, body mass index (calculated as weight in kilograms divided by height in meters squared); DCIS, ductal carcinoma in situ; ER, estrogen receptor; IDC, invasive ductal carcinoma; ILC, invasive lobular carcinoma; LCIS, lobular carcinoma in situ; NA, not applicable; PR, progesterone receptor.

^a^ Univariate linear regression analysis of patient, cancer, and treatment factors was performed on the training cohort data set.

^b^ Multivariate linear regression analysis of all patient, cancer, and treatment factors with *P* < .10 on univariate analysis was conducted. Estimates (with 95% CIs) and *P* values are displayed for the statistically significant factors in the final model.

On multivariate linear regression, only 5 variables remained, including 3 patient factors (age, BMI, and mammographic breast density), 1 cancer factor (number of pathological lymph nodes), and 1 treatment factor (ALND) (Table 2). The final equation for predicting the volume of lymphedema was as follows:

Lymphedema volume = −329 + [4 × Age] + [10 × BMI] – [37 × Mammographic Breast Density] + [13 × No. of Pathological Lymph Nodes] + [99 × ALND Treatment Use]

Higher predicted values of volume indicated greater severity of lymphedema. Negative values were indicative of a decreased likelihood of developing any lymphedema. To understand the improvements to the multivariate linear regression model with each variable, we used the Akaike information criterion (AIC) as an indicator of model quality. We found that the full model (including all 5 variables) was associated with the best performance (AIC = 1601) compared with models without age (AIC = 1605), BMI (AIC = 1610), mammographic breast density (AIC = 1602), number of pathological lymph nodes (AIC = 1610), or ALND (AIC = 1629). Correlations between variables are listed in eTable 1 in the Supplement. For example, BMI was correlated with age (Spearman correlation coefficient, 0.24; *P* < .001) and vascular disease (Spearman correlation coefficient, 0.31; *P* < .001) but not ALND (Spearman correlation coefficient, 0.10; *P* = .07). Breast density was correlated with age (Spearman correlation coefficient, -0.33; *P* < .001) and vascular disease (Spearman correlation coefficient, −0.18; *P* = .001). Age was also correlated with vascular disease (Spearman correlation coefficient, 0.48; *P* < .001) (eTable 1 in the Supplement).

**Figure 2.**
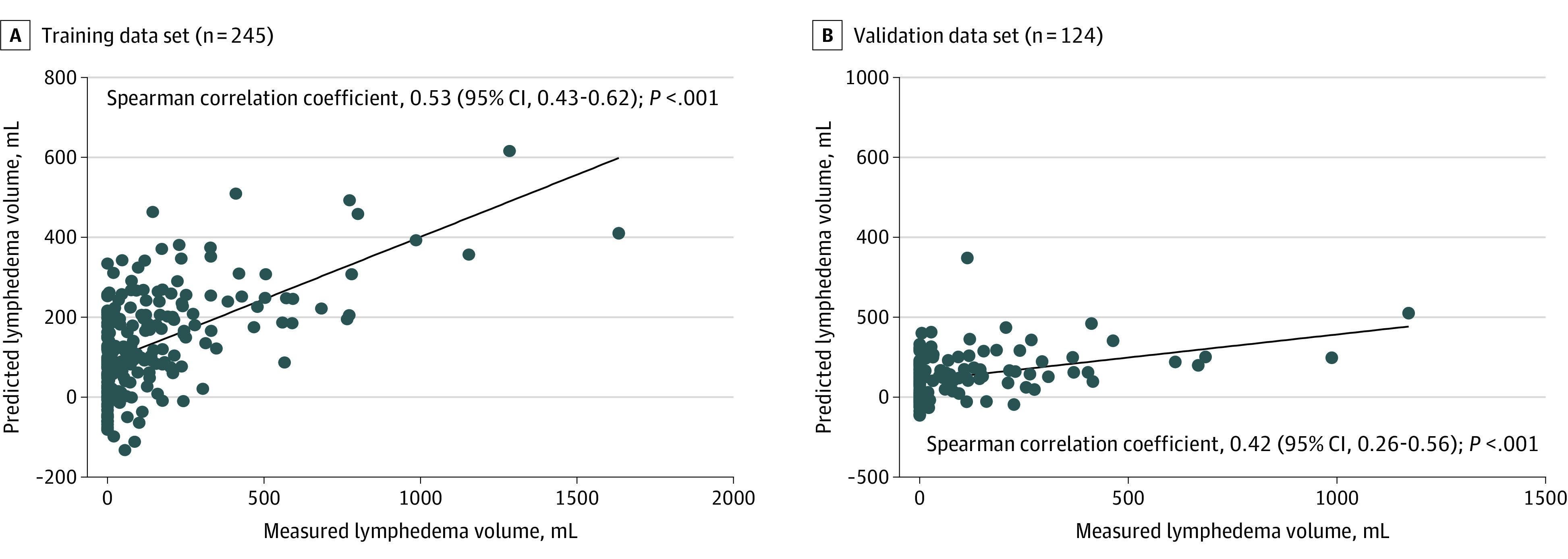
Correlation Between Measured and Predicted Lymphedema Volume Simple linear regression analysis was applied to generate a line of best fit. Two patients from each of the training and validation data sets were excluded because of missing number of pathological lymph nodes.

Comparing measured and predicted values of upper extremity lymphedema volume, Spearman correlation testing revealed a statistically significant, moderate correlation in the training cohort data set (Spearman correlation coefficient, 0.53; 95% CI, 0.43-0.62; *P* < .001). This statistically significant, moderate correlation was confirmed in the validation cohort data set (Spearman correlation coefficient, 0.42; 95% CI, 0.26- 0.56; *P* < .001) (Figure 2). The distribution of error values further verified that more than three-quarters of predictions were within 200 mL of the measured value (eFigure 1 in the Supplement).

**Figure 3.**
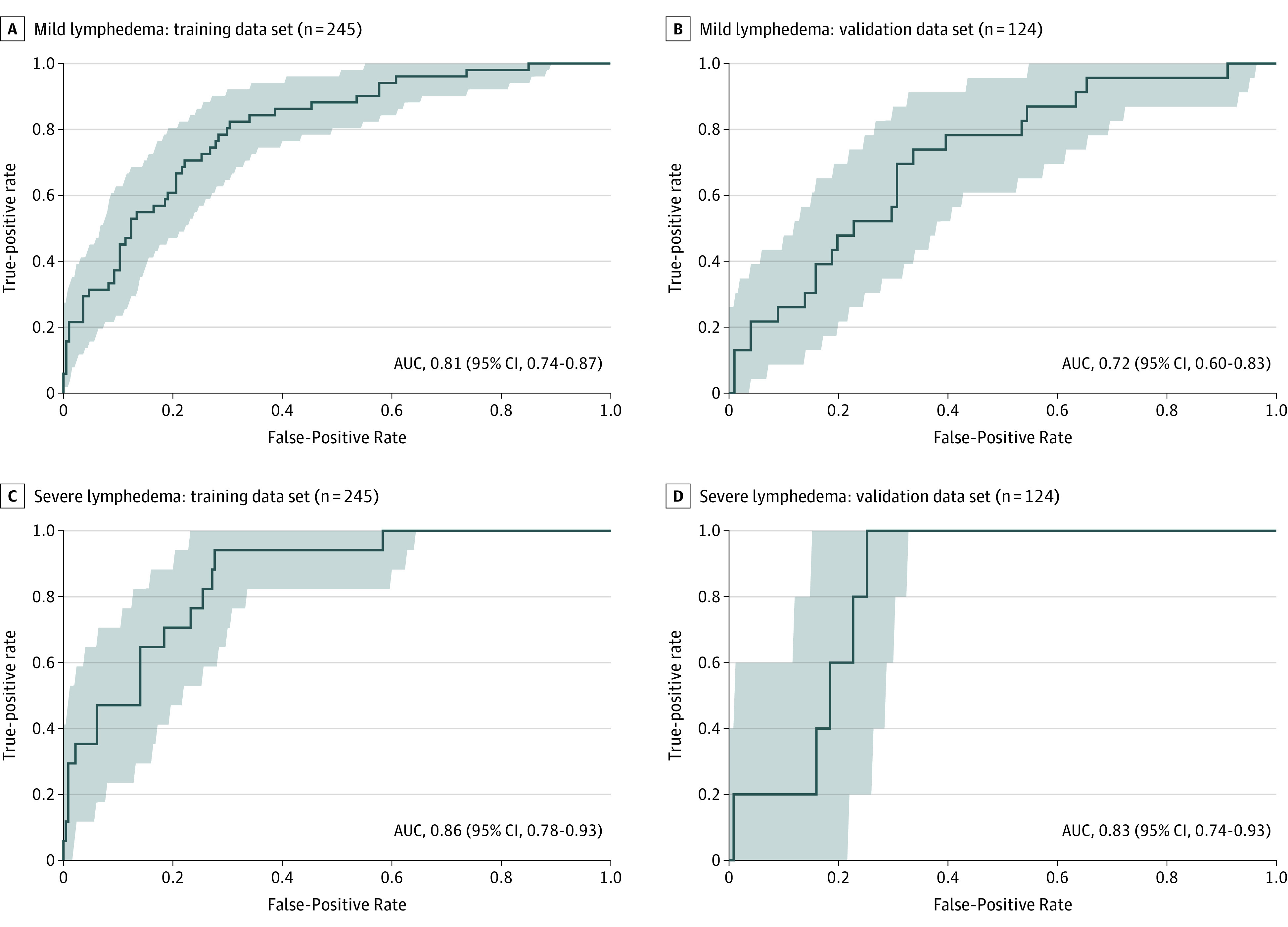
Receiver Operating Characteristic Curves for Prediction of Mild Lymphedema and Severe Lymphedema Prediction of mild lymphedema is defined by an arm volume difference of greater than 200 mL. Area under the curve (AUC) values with 95% CI are shown for the training data set (A) and for the validation data set (B). Prediction of severe lymphedema is defined by an arm volume difference of greater than 500 mL. The AUC values with 95% CIs are shown for the training data set (C) and the validation data set (D). Two patients from each of the training and validation data sets were excluded because of missing number of pathological lymph nodes.

Receiver operating characteristic curves were generated for the multivariate linear regression prediction model; the established sensitive and specific volume threshold definitions were greater than 200 mL for mild lymphedema and greater than 500 mL for severe lymphedema.^25,26^ The AUC values for predicting at least mild lymphedema were 0.81 (95% CI, 0.74-0.87) in the training cohort and 0.72 (95% CI, 0.60-0.83) in the validation cohort. For severe lymphedema, the AUC values were 0.86 (95% CI, 0.78-0.93) in the training cohort and 0.83 (95% CI, 0.74-0.93) in the validation cohort (Figure 3).

The 2-year rate of lymphedema-free survival was 58% (95% CI, 46%- 74%) in those with greater than 200 mL predicted volume (higher volume) vs 91% (95% CI, 85%-97%; *P* < .001) in those with 200 mL or less predicted volume (lower volume) (eFigure 2 in the Supplement). The HR of developing lymphedema greater than 200 mL in patients with high-volume prediction was 7.47 (95% CI, 3.92-14.21; *P* < .001) in the training cohort. Similarly, in the validation cohort, the 2-year rate of lymphedema-free survival was 68% (95% CI, 53%-88%) in those with higher predicted volume vs 83% (95% CI, 74%-93%; *P* < .41) in those with lower volume prediction (HR, 2.39; 95% CI, 1.01-5.68; *P* = .048) (eFigure 2 in the Supplement).

We constructed a matrix of the distribution of cases by mammographic breast density and volume of lymphedema (eFigure 3 in the Supplement), which demonstrated an association between fatty breasts (lowest density) and lymphedema severity (correlation coefficient, 0.998; 95% CI, 0.886-1.000; *P* = .002). Conversely, we observed an association between extremely dense breasts (highest density) on the mammogram with volume of lymphedema (correlation coefficient, –0.910; 95% CI, –0.998 to 0.412; *P* = .09). When examining the time to lymphedema development, we found a consistent pattern toward worse event-free survival probabilities in fattier (less dense) breasts, although this finding was not statistically significant (eFigure 4 in the Supplement). The 2-year lymphedema event-free survival probabilities for most dense to least dense breasts were as follows: strata 4: 58% (95% CI, 40%-84%), strata 3: 56% (95% CI, 47%-66%), strata 2: 44% (95% CI, 36%-55%), and strata 1: 49% (95% CI, 29%-82%) (*P* = .13).

To interpret the generalizability of these data, which were based on participants in the CRS Program, we compared these patients’ baseline characteristics with those of patients in the general breast oncology population at the Princess Margaret Cancer Centre (eTable 2 in the Supplement). No statistically significant differences were observed in the 5 variables used in the model (age, BMI, mammographic breast density, number of pathological lymph nodes, and ALND), suggesting that the observations from this study could be applied to the broader population of patients with breast cancer. For example, the 5-factor prediction model could be applied to those who were not referred to the CRS Program to predict lymphedema risk.

## Discussion

This prognostic study identified that the severity of lymphedema can be estimated using 5 clinical factors: (1) mammographic breast density, (2) BMI, (3) age, (4) number of pathological lymph nodes, and (5) ALND. This model underscores that lymphedema is a multifactorial disease involving patient, cancer, and treatment factors. To our knowledge, this study is the first to not only report the use of diagnostic mammographic breast density as a prognostic factor for lymphedema risk but also provide volumetric estimates of lymphedema severity.

In this study, a new prognostic patient factor for lymphedema was identified: mammographic breast density, a more direct measure of adiposity than BMI, which is calculated from height and weight. Mammography is currently the standard of care for breast cancer screening and diagnosis; thus, these data are readily available for inclusion in risk modeling. The study results revealed that fatty breasts cooccurred with worse lymphedema, and on multivariate analysis, breast density added an independent prognostic value beyond BMI.

Lymphedema is a disorder of chronic, excess adipose deposition and dysfunctional lymphatic vasculature. Thus, the mechanistic association between breast density and lymphedema risk may be explained by its reflection of (1) disrupted adipose homeostasis and (2) vascular compromise. Susceptibility to increased adipose deposition can be conveyed through the breast density metric given that it has been used as an imaging surrogate for patterns of fat distribution (eg, waist circumference and waist-hip ratios)^27,28,29,30^ and whole-body adiposity.^14,15^ This theory is further supported biologically by several studies that have identified associations between fatty breasts and metabolic syndrome, a disorder related to fat storage,^31,32^ as well as metabolic syndrome components, including elevated fasting plasma glucose,^31^ cholesterol,^33^ blood pressure,^31^ and diabetes.^34^ Furthermore, impaired fat metabolism has been shown to hinder lymphatic growth and development.^35,36^ Compromised lymphatic vasculature can lead to decreased lymphatic transport and increased severity of edema.^12,13^ This study supports the association between breast density and vascular compromise with its correlation with age (Spearman correlation coefficient, –0.33; *P* < .001), a major risk factor for vascular disease,^37^ and medical history of vascular disease (Spearman correlation coefficient, –0.18; *P* = .001) (eTable 1 in the Supplement).

As discussed earlier, BMI has been previously established as a patient risk factor for lymphedema.^7,11,38,39^ A systematic review and meta-analysis of 14 studies determined that a higher BMI carried a relative risk of 5.5.^1^ Substantial heterogeneity (*I*^2^ = 84%^11^), however, has been observed across studies, which is potentially attributable to different definitions of a high BMI (≥25 or ≥30) and binary thresholding of the BMI parameter above or below specific thresholds. In the current study, the prognostic value of a continuous BMI in predicting lymphedema severity was demonstrated, corroborating the association of BMI with lymphedema. Similar to breast density, BMI is associated with lymphedema risk, and this association can be explained by its reflection of disrupted adipose homeostasis and vascular dysfunction. Obesity, characterized by high BMI, has been associated with increased inflammation, decreased lymphatic drainage, and adipose hypertrophy.^40,41^ In this analysis, BMI was statistically significantly correlated with age (Spearman correlation coefficient, 0.24; *P* < .001) and vascular disease (Spearman correlation coefficient, 0.31; *P* < .001) (eTable 1 in the Supplement), indicating BMI’s association with vascular compromise. Body mass index appears to reflect more disease-related vascular compromise compared with breast density, which has a higher correlation with age-related vascular changes; thus, BMI and breast density metrics can be complementary. Previous studies have reported a higher number of failed sentinel lymph node biopsies among patients with obesity,^42^ thereby requiring completion ALND, which is associated with higher lymphedema risk. Other studies have countered this observation, suggesting that obesity does not have a substantial implication for the success of sentinel lymph node biopsies.^43,44^ The current study supports the findings of these studies in that we did not observe a correlation between BMI and greater use of ALND (Spearman correlation coefficient, 0.10; *P* = .07) (eTable 1 in the Supplement).

Age was also identified on multivariate analysis as an independent patient risk factor for lymphedema severity. Age is among the most examined factors in lymphedema risk modeling because of its mandatory documentation in clinical records. Despite its correlation with lymphedema in some studies,^45,46,47^ a systematic review and meta-analysis of 7 studies reported an odds ratio ranging from 0.4 to 3.3, indicating inconclusive evidence of the association of age with lymphedema.^1^ Furthermore, another review of 12 studies reported the high heterogeneity score of age (*I*^2^ = 66%).^11^

As mentioned, older age is a main risk factor of vascular diseases,^37^ such as hypertension, which has been associated with lymphedema.^4,48,49,50^ These observations are supported by data from this current study, which observed a moderate correlation between age and vascular comorbidities (Spearman correlation coefficient, 0.48; *P* < .001) (eTable 1 in the Supplement), which encompassed the diagnoses of hypertension,^11,38,39,51^ hypercholesterolemia,^12,13,52^ and diabetes.^11,38,53^ High cholesterol levels have been mechanistically associated with the degeneration of lymphatic vessels, which has led to decreased lymphatic transport, increased leakiness, and increased edema.^12,13^ Elevated glucose levels have similarly been observed with decreased lymphatic clearance of interstitial macromolecules during obesity.^40,41^ Age, however, is not a perfect surrogate for vascular health and lymphedema risk and was complemented by other metrics such as mammographic breast density and BMI, which were all prognostic on multivariate analysis.

Well-established cancer and treatment factors were corroborated in this study. On multivariate analysis, the number of pathological lymph nodes had statistically significant prognostic value for lymphedema severity. This parameter is a surrogate for the extent of metastatic^54^ and treatment-related damage to the lymphatic vasculature and is well described in the literature.^11,55,56,57^ Similarly, ALND is another measure for the greater extent of surgical damage to the lymphatics and has been confirmed in multiple studies.^11,58,59,60^

Overall, the multivariate model will allow for the risk stratification of patients into 4 clinically relevant groups: those who are (1) unlikely to develop any lymphedema, (2) likely to develop limited lymphedema (≤200 mL), (3) likely to develop mild lymphedema (>200 and ≤500 mL), and (4) likely to develop severe lymphedema (>500 mL). Patients who are unlikely to develop lymphedema can undergo routine follow-up care. For patients who are likely to develop limited or mild lymphedema, early modification of risk factors (eg, exercise and BMI reduction), conservative use of ALND, and/or more frequent monitoring for lymphedema development will facilitate early initiation of conservative therapies (eg, compression therapies) that minimize morbidity from lymphedema. Patients who are likely to develop severe lymphedema, in addition to following the recommendations for other groups, may be candidates for surgical therapies (eg, lymphovenous bypass) in which early interventions are more successful when performed before the development of fibrosis, a characteristic of chronic lymphedema.^61^

### Limitations and Strengths

This study has both limitations and strengths. The main limitation is that the data were extracted retrospectively. A major strength, however, is that all patients were followed up in a single clinic in which the visits were uniformly conducted and the information was recorded prospectively by trained clinic personnel using a standard dictation template. Furthermore, missing data were imputed for 2 variables during modeling. The primary outcome of association with lymphedema severity, however, remained consistent when analyzed without imputation. In addition, the study follow-up allowed for the inclusion of most upper extremity lymphedema diagnoses given that more than 70% of patients with breast cancer experience onset of lymphedema within a year.^7^ Specifically, in the present cohort, the median follow-up time was 22 months for participants who were classified as having no lymphedema. Truncal (breast and chest wall) lymphedema was not included because it was not amenable to reproducible quantification. To ascertain the applicability of this model to patients in the general breast oncology population, we compared the patients referred to the CRS Program with patients in the general breast oncology population, and we observed no statistically significant difference in baseline characteristics pertaining to the model parameters, suggesting the potential generalizability of the findings to the broader breast oncology population. Further validation at other centers is required to confirm the utility of the model, which could be easily conducted given the availability of mammography.

## Conclusions

This study identified 5 readily available clinical factors, including patient, cancer, and treatment factors, that can be used to generate volumetric estimates of lymphedema severity in patients with breast cancer. To our knowledge, this study is the first to use mammographic breast density as an independent prognostic factor for lymphedema risk and to provide volumetric estimates of lymphedema morbidity. Predictions of lymphedema occurrence and morbidity can help triage patients for increased disease monitoring and thus allow for earlier diagnosis and optimal management of this condition. Such predictions also can assist in risk stratification in future clinical trials on novel therapeutic interventions for lymphedema.

## Notes

Supplement. **eFigure 1.** Distribution of Errors Between Measured and Predicted Lymphedema Volumes **eFigure 2.** Prediction of Lymphedema Control by Lymphedema Volume in Training and Validation Data Sets **eFigure 3.** Distribution of Mammographic Breast Density and Lymphedema Severity Among Breast Cancer Patients **eFigure 4.** Prediction of Lymphedema Control by Breast Density **eTable 1.** Evaluation of Correlation Between Variables **eTable 2.** Comparison of the Study Population to the General Breast Oncology Clinic Population
