# Supplementary material for: Development and Validation of a Risk Model for Breast Cancer–Related Lymphedema
Source: JAMA Netw Open. 2020 Nov 11;3(11):e2024373. doi: 10.1001/jamanetworkopen.2020.24373 (PMC7658732; doi:10.1001/jamanetworkopen.2020.24373)
Supplement: Supplement. — eFigure 1. Distribution of Errors Between Measured and Predicted Lymphedema Volumes eFigure 2. Prediction of Lymphedema Control by Lymphedema Volume in Training and Validation Data Sets eFigure 3. Distribution of Mammographic Breast Density and Lymphedema Severity Among Breast Cancer Patients eFigure 4. Prediction of Lymphedema Control by Breast Density eTable 1. Evaluation of Correlation Between Variables eTable 2. Comparison of the Study Population to the General Breast Oncology Clinic Population [file jamanetwopen-e2024373-s001.pdf]

Supplementary Online Content

Kwan JYY, Famiyeh P, Su J, et al. Development and validation of a risk model for breast cancer–related lymphedema. *JAMA Netw Open*. 2020;3(11):e2024373. doi:10.1001/jamanetworkopen.2020.24373

eFigure 1. Distribution of Errors Between Measured and Predicted Lymphedema Volumes

eFigure 2. Prediction of Lymphedema Control by Lymphedema Volume in Training and Validation Data Sets

eFigure 3. Distribution of Mammographic Breast Density and Lymphedema Severity Among Breast Cancer Patients

eFigure 4. Prediction of Lymphedema Control by Breast Density

eTable 1. Evaluation of Correlation Between Variables

eTable 2. Comparison of the Study Population to the General Breast Oncology Clinic Population

This supplementary material has been provided by the authors to give readers additional information about their work.

©2020 Kwan JYY et al. *JAMA Network Open*

eFigure 1. Distribution of Errors Between Measured and Predicted Lymphedema Volumes

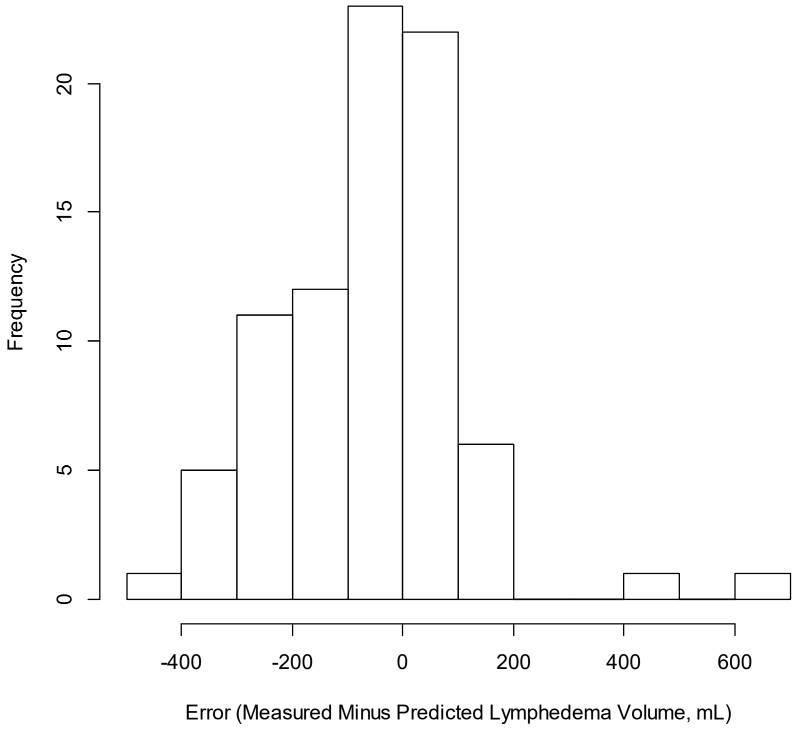

The distribution of errors between the measured and predicted lymphedema volumes in the validation cohort are shown. The frequency of occurrence is on the y-axis and error is on the x-axis. The majority of the predicted values are similar to the measured values as shown by the proximity of the data to zero.

©2020 Kwan JYY et al. *JAMA Network Open*

eFigure 2. Prediction of Lymphedema Control by Lymphedema Volume in Training and Validation Data Sets

A

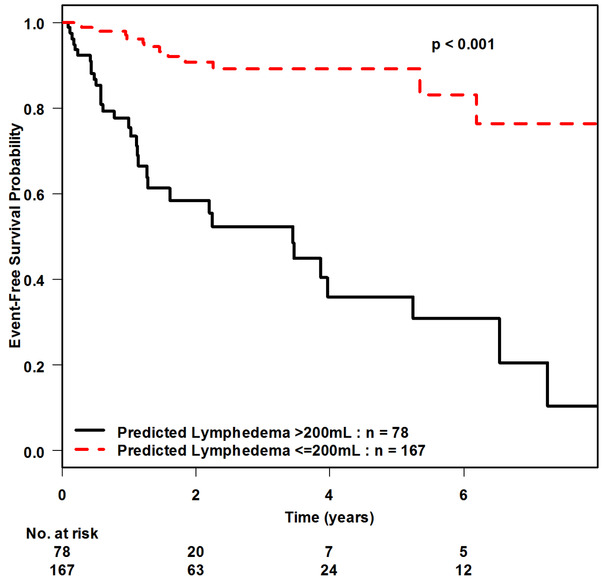

Strata	Events/total	1-year	2-year
Total	51 / 245	89% (85-94)	81% (75-87)
Predicted Lymphedema ≤200 mL	17 / 167	96% (93-100)	91% (85-97)
Predicted Lymphedema >200 mL	34 / 78	76% (66-87)	58% (46-74)

B

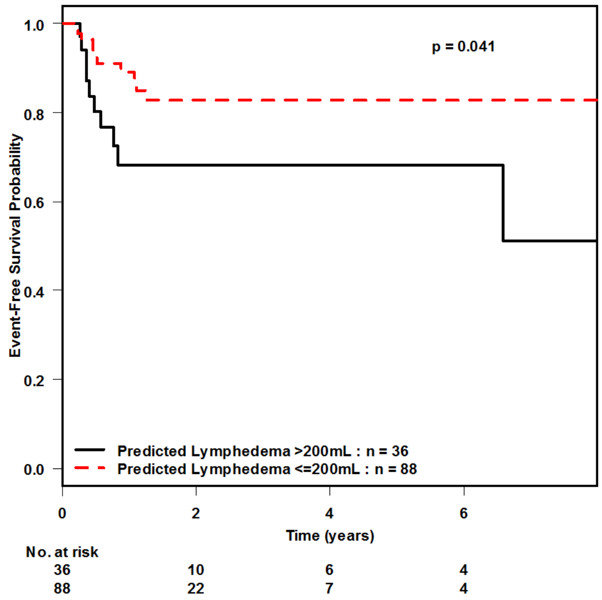

©2020 Kwan JYY et al. *JAMA Network Open*

Strata	Events/total	1-year	2-year
Total	23 / 124	83% (76-91)	79% (71-88)
Predicted Lymphedema ≤200 mL	11 / 88	89% (82-97)	83% (74-93)
Predicted Lymphedema >200 mL	12 / 36	68% (53-88)	68% (53-88)

Kaplan-Meier event-free probability graphs were generated from the initial breast cancer surgery date to the occurrence of lymphedema or last follow-up appointment. Events were demarcated using the conservative definition of at least mild lymphedema (>200 mL volumetric difference between arms). Two risk groups were defined based on the predicted volumes of lymphedema calculated from the model. High volume predictions were significantly associated with increased lymphedema risk in the: (A) training (Hazards Ratio (HR) 7.47, 95% CI 3.92-14.21, $p<0.001$); and (B) validation cohorts (HR 2.39, 95% CI 1.01-5.68, $p<0.05$). Event numbers and lymphedema-free survival at one- and two-years post-surgery rates (and 95% CI) are shown in the accompanying Tables. Two patients from each of the training and validation data sets were excluded to due to missing pathologic lymph node numbers. Kaplan-Meier p-values for differences between groups are shown on the graphs. Hazard ratios were calculated using Cox proportional hazards regression.

eFigure 3. Distribution of Mammographic Breast Density and Lymphedema Severity Among Breast Cancer Patients

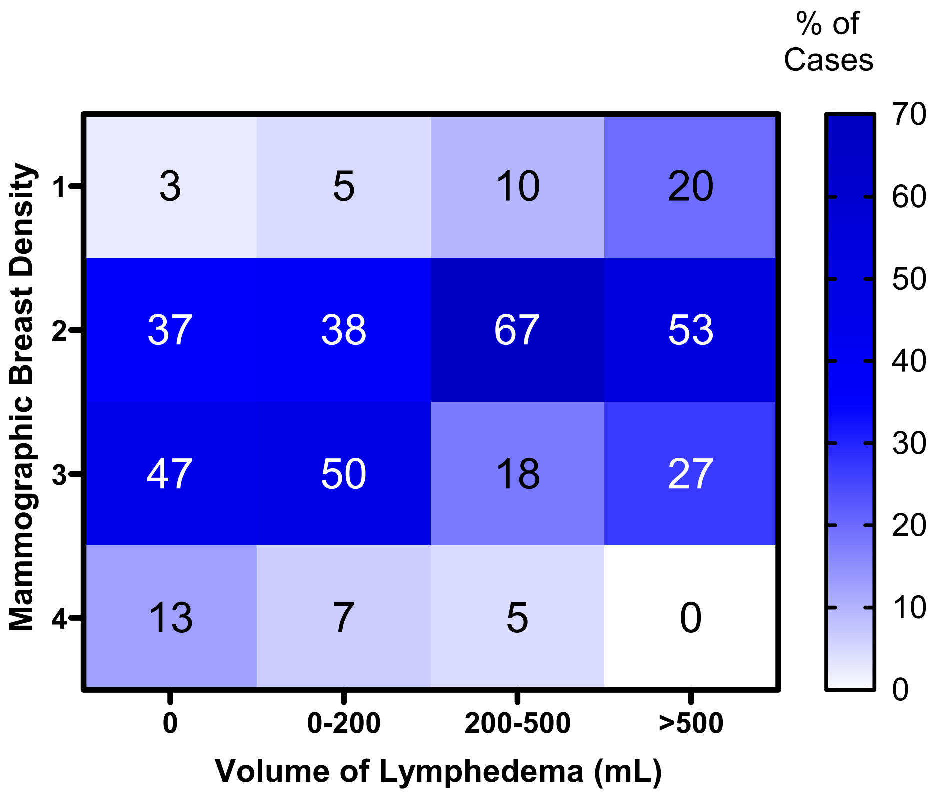

The distribution of the percentages of cases of each BI-RADS (Breast Imaging Reporting and Database System) breast density is shown as a function of lymphedema volume outcome. Increased proportion of fatty (low-density) breasts were noted among patients with severe lymphedema; conversely, patients with extremely dense breasts comprised a smaller proportion of patients with more severe lymphedema.

©2020 Kwan JYY et al. *JAMA Network Open*

eFigure 4. Prediction of Lymphedema Control by Breast Density

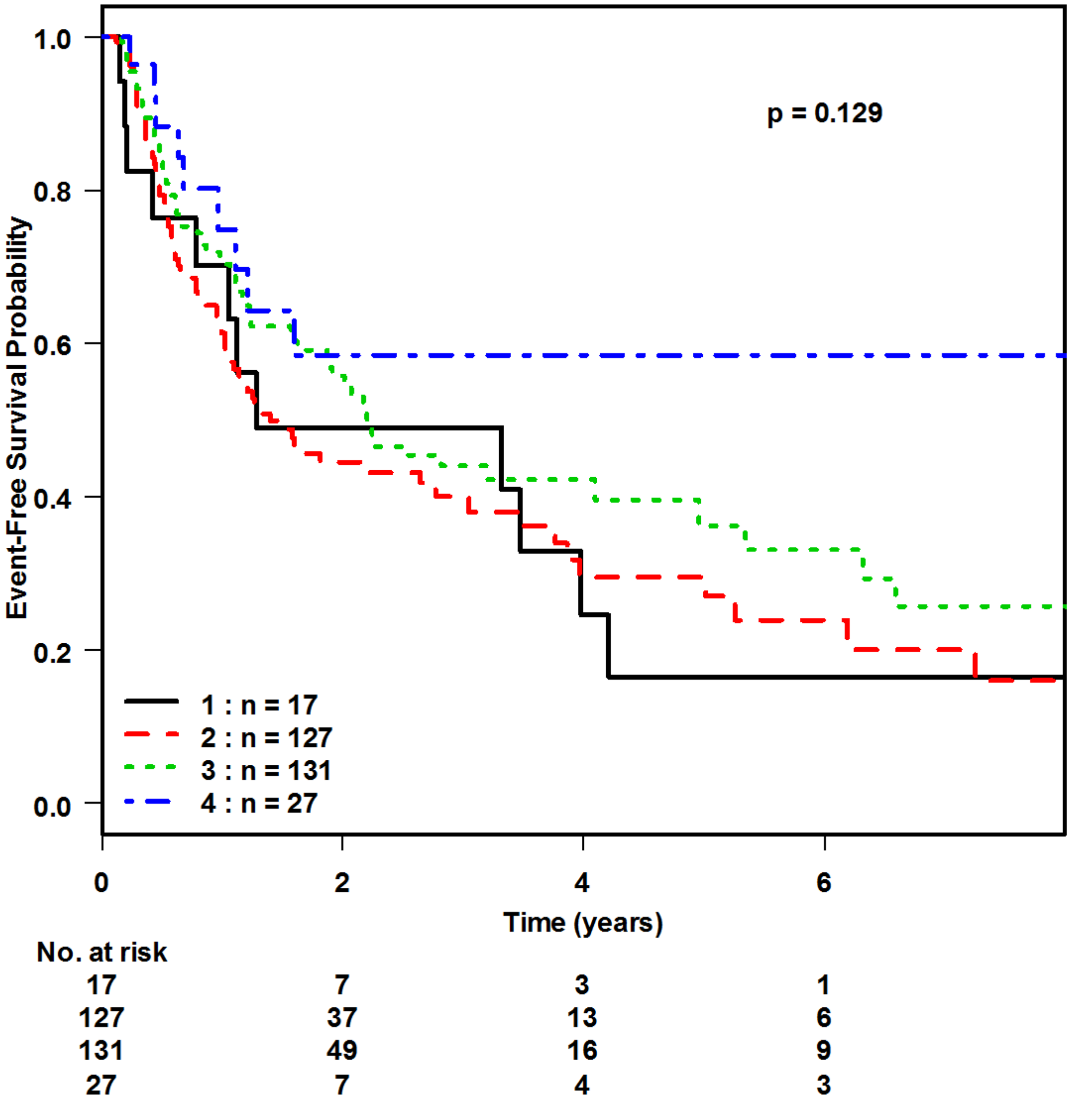

Strata	Events/total	1-year	2-year
1	13 / 17	70% (51-96)	49% (29-82)
2	79 / 127	61% (53-71)	44% (36-55)
3	71 / 131	71% (64-79)	56% (47-66)
4	10 / 27	75% (59-95)	58% (40-84)

Kaplan-Meier event-free probability graphs were generated from the initial breast cancer surgery date to the occurrence of lymphedema or last follow-up appointment. Events were demarcated by any lymphedema of greater than zero volume. Risk groups were defined by mammographic breast densities of 1 (fatty) to 4 (dense). No imputed density values were used in this graph. Kaplan-Meier p-value for differences between groups are shown on the graph. There is a trend towards worse event-free survival probabilities among those with more fatty breasts. Event numbers and lymphedema-free survival at one- and two-years post-surgery rates (and 95% CI) are shown in the accompanying Table.

©2020 Kwan JYY et al. *JAMA Network Open*

eTable 1. Evaluation of Correlation Between Variables

Spearman correlation coefficients were calculated among the following patient, cancer, and treatment factors, including all investigative patient factors that were significant on univariate and multivariate analyses as well as all established cancer and treatment factors identified on multivariate analysis. Correlation coefficient, p-value, and sample number (n) are displayed for each test. Abbreviations: BMI = Body Mass Index; ALND = Axillary Lymph Node Dissection.

		PATIENT			CANCER	TREATMENT
		**History of Vascular Disease**	**BMI**	**Breast Density**	**Number of Pathologic Lymph Nodes**	**ALND**
**Age**	Coefficient	0.475	0.237	-0.330	0.031	0.023
	P-value	<0.0001	<0.0001	<0.0001	0.550	0.660
	n	373	312	302	369	373
**History of Vascular Disease**	Coefficient		0.308	-0.183	0.101	0.055
	P-value		<0.0001	0.0014	0.053	0.293
	N		312	302	369	373
**BMI**	Coefficient			-0.429	-0.065	0.103
	P-value			<0.0001	0.255	0.071
	n			263	310	312
**Breast Density**	Coefficient				0.008	-0.080
	P-value				0.895	0.166
	n				299	302
**Number of Pathologic Lymph Nodes**	Coefficient					0.528
	P-value					<0.0001
	n					369

©2020 Kwan JYY et al. *JAMA Network Open*

eTable 2. Comparison of the Study Population to the General Breast Oncology Clinic Population

The study population (i.e. patients referred to the Cancer Rehabilitation and Survivorship (CRS) Program) were compared to a consecutive sampling of the general breast oncology patient population (i.e. not referred to the CRS) over the same follow-up time period. Non-lymphedema patients of the study population were used for comparison since almost all lymphedema patients were referred to the survivorship clinic. Baseline patient, disease, and treatment characteristics are presented using mean (with standard deviation) and median (with minimum, maximum) for continuous variables and frequency (with percentages) for categorical variables. Group comparisons were performed using a t-test (continuous variables) or chi-square test (categorical variables). No significant differences were observed for the five clinical characteristics of the model.

Characteristic	Referred Population: Cancer Rehabilitation and Survivorship Program Non-Lymphedema Patients (n=147)	Non-Referred Population: General Breast Oncology Clinic Non-Lymphedema Patients (n=67)	P-value
**PATIENT FACTORS**			
**Age**			0.23
Mean (Standard Deviation)	51.9 (9.2)	53.7 (11.8)	
Median (Min,Max)	51.2 (32.1,79.2)	51.5 (29.7, 82.5)	
**Body Mass Index (BMI)**			0.36
Mean (SD)	25.8 (5.1)	26.6 (6.5)	
Median (Min,Max)	25.0 (11.0,42.1)	25.3 (18.2,45.1)	
Missing	21	7	
**Mammographic Breast Density**			0.49
Mean (Standard Deviation)	2.7 (0.7)	2.6 (0.9)	
Median (Min,Max)	3 (1,4)	2.5 (1,4)	
Missing	18	33	
**CANCER FACTOR**			
**Number of Pathologic Lymph Nodes**			
Mean (SD)	1.2 (2.4)	1.1 (2.8)	0.79
Median (Min,Max)	0 (0,18)	0 (0,16)	
Missing	2	0	
**TREATMENT FACTOR**			
**Axillary Lymph Node Dissection**	35 (24)	15 (22)	0.96

©2020 Kwan JYY et al. *JAMA Network Open*
